# Clinical analysis and predictive factors associated with improved visual acuity of infectious endophthalmitis

**DOI:** 10.1186/s12886-020-01517-7

**Published:** 2020-06-29

**Authors:** Zhao Gao, Yunda Zhang, Xiaohong Gao, Ximei Zhang, Tao Ma, Gaiyun Li, Jingjing Wang, Hua Yan

**Affiliations:** 1 Department of Ophthalmfology, Tianjin Medical University General Hospital, No. 154, Anshan Road, Tianjin, 300052 China; 2 Department of Vitreoretinopathy, Shanxi Eye Hospital, Taiyuan, 030001 Shanxi China; 3 Department of Medical Services, Shanxi Eye Hospital, Taiyuan, 030001 Shanxi China

**Keywords:** Endophthalmitis, Clinical characteristics, Causative organisms, Visual acuity, Predictive factors

## Abstract

**Background:**

To describe the clinical characteristics and analyze the predictive factors associated with improved visual acuity of 359 patients with infectious endophthalmitis.

**Methods:**

This study retrospectively analyzed 359 eyes of 359 patients with infectious endophthalmitis from January 2014 to December 2018. The findings summarized some epidemiological characteristics of these patients, including age, sex, occupation, patient visit time, etiology, causative organisms, therapy, and best-corrected visual acuity. Multivariate logistic regression was performed to predict the relative factors of improved visual acuity (VA).

**Results:**

Overall, 283 (78.83%) patients were male. The mean age was 48.0 ± 18.27 years. Ocular trauma, especially open globe injuries (246, 68.5%) was the most common etiology of infectious endophthalmitis in this study. The etiologies of infectious endophthalmitis were open globe injuries (68.5%), intraocular surgery (22.6%), and corneal ulcer-associated (6.7%) and endogenous causes (2.2%). In the etiology classification and visual acuity improvement group, had statistically significant differences in factors such as age, sex, patient visit time, pre-therapy visual acuity, etc. The average Logarithm of the Minimum Angle of Resolution (logMAR) best-corrected visual acuity on pre-therapy was 2.28 ± 0.60, and it had significantly improved to 1.67 ± 0.83 post-therapy (*P* < 0.05). Logistic regression analysis showed that visit time > 7 day (*P* = 0.034, OR = 0.522, 95% CI: 0.286–0.953), pre-therapy VA ≦logMAR 2.3 (*P* = 0.032, OR = 1.809, 95% CI: 1.052–3.110), post-surgical (vs. posttraumatic; *P* = 0.023, OR = 2.100, 95% CI: 1.109–3.974), and corneal ulcer-associated etiologies (vs. posttraumatic; *P* = 0.005, OR = 0.202, 95%CI: 0.066–0.621) were significantly associated with improved visual acuity after adjusting for possible confounding factors.

**Conclusions:**

Among the patients with infectious endophthalmitis, middle-aged male, especially farmers and workers, accounted for a large proportion. Open globe injuries were the main cause and the gram-positive bacteria were the major causative organisms. The final visual outcomes seemed to vary according to the type of endophthalmitis, but early treatment and good initial visual acuity were important factors for visual acuity improvement.

## Background

Endophthalmitis is a severe inflammation of intraocular fluids usually caused by the infection of contaminating microorganisms following trauma, surgery, or hematogenous spread from the distant infection parts. Because of the difficulty in diagnosis and the low bacterial culture positivity rate, the detection and treatment of this disease are primarily based on the physician’s clinical experience [1–3]. Although the prognosis of endophthalmitis has shown enormous progress in the cases of endophthalmitis due to the use of effective intraocular antibiotics and the advances in vitreoretinal surgery, there are still several cases of virulent infection leading to irreversible visual impairment and even the enucleation of the eye [4]. Few studies have investigated the risk factors associated with endophthalmitis and visual impairment. Durand reported that there was a reasonably high correlation between the visual outcome and pathogenic microbiology [5], *Streptococci* can produce severe endophthalmitis with a poor visual outcome, whereas coagulase-negative *staphylococci* cause milder endophthalmitis in general. Yosanan et al. found that the only possible predictive factor associated with improved visual outcomes was pars plana vitrectomy (PPV) within 3 days [6].

Endophthalmitis can be classified as exogenous and endogenous. Exogenous endophthalmitis is categorized as postsurgical, posttraumatic, or corneal ulcer-associated. In contrast, endogenous endophthalmitis is caused by blood infection or immunosuppression [7, 8]. The incidence of endophthalmitis varies according to location, economy, and ethnicity. In a German study, Lothar Kraus et al. reported that endophthalmitis following open ocular injury accounted for 12% of the examined patients with endophthalmitis, while 41% of the patients showed endogenous endophthalmitis [9]. In contrast, a western China study reported that only 7.8% of 1593 endophthalmitis cases had an endogenous origin, and up to 82.6% were posttraumatic in nature [2].

Moreover, endophthalmitis has a high incidence in middle-aged male patients [2], which could have a significant impact on the patients’ family and society. Therefore, it is extremely important to thoroughly understand the epidemiological characteristics of endophthalmitis and the predictable factors associated with visual acuity (VA) improvement.

## Methods

### Ethical approval

This retrospective, single-center study was conducted in accordance with the tenets of the Declaration of Helsinki of the World Medical Association, and approved by the institutional review board of Shanxi Eye Hospital. The requirement for informed consent was waived due to the retrospective nature of this study.

### Participants

Patients who were diagnosed with endophthalmitis between January 1, 2014, and December 31, 2018, from Shanxi eye hospital, were included in this study. The included criteria were as follows: decreased vision, red eye and pain associated with hypopyon, fibrin, severe anterior chamber reaction, vitreous inflammation hypopyon, decreased red reflex, and history of intraocular surgery or an open eye injury or systemic disease [10]. The excluded criteria were as follows: allergic uveitis of the lens cortex, sympathetic ophthalmia, toxic reaction syndrome of the anterior segment after intraocular surgery, various forms of autoimmune uveitis, and other forms of uveitis. There were 359 eyes (359 patients) were ultimately included in the final statistical analysis.

### Measurements

Patient data included the time of injury (grouped by year), age (0–15, 16–30, 31–45, 46–60, 61–75, or ≥ 76 years), sex (male or female), marital status (married, single, divorced, or widowed), occupation (farmer, worker, office clerk, retired, student, or others), etiology (posttraumatic, corneal ulcer-associated, endogenous and postsurgical, including post-cataract, post-glaucoma, post-PPV, post- intraocular injection (IVI)), pre-therapy and post-therapy VA, therapy modalities (medical therapy, intravitreal antibiotic injections, pars plana vitrectomy, and enucleation), and causative organisms (Gram-positive, Gram-negative, fungi, or culture negative).

VA was converted to logMAR units using an international standard visual chart. Counting fingers (CFs), hand movement (HM), light perception (LP), and no LP (NLP) were converted to 1.9, 2.3, 2.7, and 3.0 logMAR, respectively [11]. Eyes that had been enucleated were assigned a logMAR value of 3.0 (NLP) [12].

The improved VA after treatment was considered the primary indicator. The patients were classified as having “improved” VA when their best-corrected VA (BCVA) rates were better post-therapy than pre-therapy. They were classified as “not improved” when the final BCVA rates were stable or worse than the initial values [6].

We followed an endophthalmitis protocol [10, 13–15]: All patients received topical and intravenous antibiotics, including vancomycin and/or ceftazidime. Then, we adjusted the antibiotic dosage according to the results of the bacterial culture and the drug sensitivity test. (1) There are inflammatory cells(++)in the anterior chamber, but no hypopyon and vitreous opacity are found, which should be closely observed and combined with medical therapy (MT), defined as the appropriate topical, periocular, and systemic antibiotic administration. (2) There is hypopyon or slight opacity of the vitreous body examined by B-ultrasound, and the fundus red light reflex can be seen. Moreover patients with VA of HM (2.3 logMAR) or better, can receive intravitreal antibiotic injections (IVA) combined with MT. Patients who presented with a VA of light perception and corneal involvement precluding surgery were managed similarly. (3) Patients with VA worse than HM (2.3 logMAR) and with sufficiently clear corneal, hypopyon complicated, vitreous opacity, and disappearance of red light reflex were treated with PPV as a primary procedure, including IVA and MT. When there was an intraocular foreign body (IOFB), PPV was performed first. (4) In cases where the aforementioned treatment is not effective, the corneal ulcer perforation is serious, and the patient’s vision has no light perception, enucleation should be considered.

### Statistical analysis

Data were aggregated using Microsoft Excel (Microsoft Corporation, Redmond, WA, USA) and statistically analyzed using SPSS 25.0 (IBM, Corp., Armonk, NY, USA). The results of descriptive analyses were expressed as counts and percentages for categorical variables, and as means ± standard deviations (SD) for continuous variables. Differences in the measurement data were detected by analyses using the t- or χ^2^ test. Multivariate analysis to ascertain the identified variables on the likelihood of improved visual outcome was performed using binomial logistic regression. The difference was considered significant when the *P*-value was < 0.05.

## Results

**Fig. 1:**
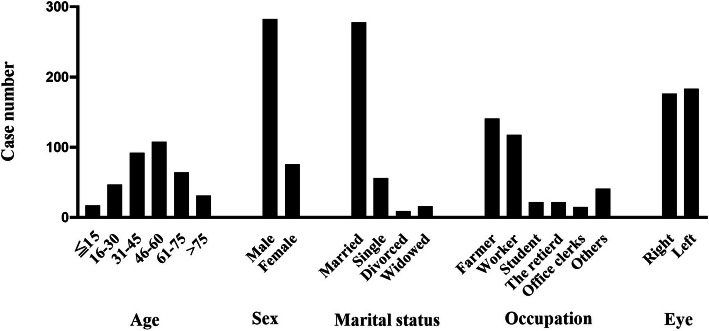
Patient characteristics with infectious endophthalmitis in the Shanxi Eye Hospital, China from 2014 to 2018

The basic characteristics of patients with infectious endophthalmitis recruited in this retrospective study are shown in Fig. 1. Altogether, 359 eyes from 359 patients were diagnosed as showing infectious endophthalmitis, and the patients were treated at our hospital within 5 years. In the 5-year case count, the overall trend showed an increase, but with a small drop in 2016 and 2017 (2014: 62, 2015: 76, 2016: 73, 2017: 68, and 2018: 80).

### Age, sex, marital status, occupation and eye characteristics

The average age of the injured patients was 48.0 ± 18.27 years (range, 4 to 86 years), and the median age was 48 years. The 46–60-year (108, 30.1%) and 31–45-year (92, 25.6%) age groups contained the most cases. Of the 359 patients, 283 were male (78.8%) and 76 were female (21.2%). The male-to-female ratio was 3.7:1. In the injured population, 77.4% (278) of the patients were married, 15.6% (56) were single, 2.5% (9) were divorced, and 4.5% (16) were widowed. The patients were mainly involved in five kinds of occupations: farmers, workers, students, retired, office clerks, and others, with the corresponding incidence rates being 39.3, 32.9, 6.1, 6.1, 4.2, and 11.4%, respectively. Among the 359 patients, 51.0% (176) and 49.0% (183) showed only unilateral right and left eye involvement, respectively, and no one showed bilateral involvement.

### Causative organisms

Following the onset of endophthalmitis, 316 patients (88.0%) underwent diagnostic tapping of ocular specimens for microbiological investigations, including vitreous and/or aqueous tap. All 316 diagnostic taps were performed at the time of initial presentation of ocular symptoms and prior to commencement of intravitreal antimicrobial therapy. The incidences of negative and positive cultures were 55.4, and 44.6%, respectively. Gram-positive organisms, Gram-negative organisms, mixed bacterial populations, and fungi were found to be the causative factors in 115 (81.6%), 16 (11.3%), four (2.8%), and six eyes (4.3%), respectively. As shown in Table 1, the most common microorganisms were *Staphylococcus epidermidis* (59 cases), followed by *S. aureus* (11 cases).

**Table 1:** Causative organisms in the 316 eyes with endophthalmitis

Causative organisms	n (316)	Rate (%)	Positive rate (%)
**Culture-positive**	141	44.62%	100.00%
Gram-positive	115	36.39%	81.56%
*S. epidermidis*	59	18.67%	41.84%
*S. aureus*	11	3.48%	7.80%
*V. Streptococci*	9	2.85%	6.38%
*S. pneumoniae*	7	2.22%	4.96%
*Enterococcus*	7	2.22%	4.96%
*S. mutans*	4	1.27%	2.84%
*T. Streptococcus*	3	0.95%	2.13%
*Micrococcus luteus*	3	0.95%	2.13%
*Bacillus spp.*	4	1.27%	2.84%
*Corynebacterium*	2	0.63%	1.42%
others	6	1.90%	4.26%
Gram-negative	16	5.06%	11.35%
*Sphingomonas*	4	1.27%	2.84%
*K. pneumoniae*	2	0.63%	1.42%
*P. aeruginosa*	2	0.63%	1.42%
*Dry Neisseria*	2	0.63%	1.42%
*S. maltophilia*	2	0.63%	1.42%
others	4	1.27%	2.84%
Mixed bacteria	4	1.27%	2.84%
fungus	6	1.90%	4.26%
**Culture-negative**	175	55.38%	

### Etiological classification

Etiological classification of endophthalmitis was demonstrated (Table 2). Table 2 shows that ocular trauma, especially open globe injury, was the most frequent cause in our cases, accounting for 68.52% of all patients, Among the 246 posttraumatic cases, 87 (35.37%) involved IOFB; 71 (28.86%) and 16 (6.50%) involving metals and non-metallic items, respectively. And 197 eyes (80.08%) had zone I injuries, followed by zone II (44,17.89%) and zone III (5, 2.03%). Post-surgical endophthalmitis was the second most common cause, accounting for 22.6%, and included post-cataract (62.96%), post-glaucoma (24.69%), post-PPV (11.11%), and post-IVI (1.23%). Other causes were corneal ulcer-associated (CA) (6.69%) and endogenous (2.22%).

**Table 2:** The etiological classification of infectious endophthalmitis

Variables	Posttraumatic	Postsurgical	Corneal ulcer-associated	Endogenous	***Statistics***	***P***
	246 (68.52%)	81 (22.56%)	24 (6.69%)	8 (2.22%)		
**Year**						
2014	44 (17.89%)	16 (19.75%)	1 (4.17%)	1 (12.50%)	χ^2^ = 10.28	0.592
2015	58 (23.58%)	14 (17.28%)	3 (12.50%)	1 (12.50%)		
2016	47 (19.11%)	19 (23.46%)	6 (25.00%)	1 (12.50%)		
2017	45 (18.29%)	16 (19.75%)	5 (20.83%)	2 (25.00%)		
2018	52 (21.14%)	16 (19.75%)	9 (37.50%)	3 (37.50%)		
**Age**	41.67 ± 15.51	62.21 ± 17.17	58.67 ± 11.94	67.13 ± 16.13	F = 43.04	< 0.0001
≦15	15 (6.10%)	2 (2.47%)	0 (0.00%)	0 (0.00%)	χ^2^ = 134.5	< 0.0001
16–30	42 (17.07%)	5 (6.17%)	0 (0.00%)	0 (0.00%)		
31–45	84 (34.15%)	3 (3.70%)	4 (16.67%)	1 (12.50%)		
46–60	80 (32.52%)	17 (20.99%)	9 (37.50%)	2 (25.00%)		
61–75	20 (8.13%)	33 (40.74%)	10 (41.67%)	2 (25.00%)		
> 75	5 (2.03%)	21 (25.93%)	1 (4.17%)	3 (37.50%)		
**Sex**						
Male	219 (89.02%)	42 (51.85%)	6 (25.00%)	4 (50.00%)	χ^2^ = 84.69	< 0.0001
Female	27 (10.98%)	39 (48.15%)	18 (75.00%)	4 (50.00%)		
**Occupation**						
Farmer	90 (36.59%)	31 (38.27%)	16 (66.67%)	4 (50.00%)	χ^2^ = 102.7	< 0.0001
Worker	108 (43.90%)	9 (11.11%)	1 (4.17%)	0 (0.00%)		
Student	20 (8.13%)	2 (2.47%)	0 (0.00%)	0 (0.00%)		
The retired	1 (0.41%)	16 (19.75%)	4 (16.67%)	1 (12.50%)		
Office clerks	10 (4.07%)	4 (4.94%)	1 (4.17%)	0 (0.00%)		
Others	17 (6.91%)	19 (23.46%)	2 (8.33%)	3 (37.50%)		
**Eye**						
Right	131 (53.25%)	48 (59.26%)	12 (50.00%)	1 (12.50%)	χ^2^ = 6.61	0.085
Left	115 (46.75%)	33 (40.74%)	12 (50.00%)	7 (87.50%)		
**Visit time**						
≦7d	209 (84.96%)	60 (74.07%)	6 (25.00%)	6 (75.00%)	χ^2^ = 47.41	< 0.0001
> 7d	37 (15.04%)	21 (25.93%)	18 (75.00%)	2 (25.00%)		
**Therapy**						
MT	13 (5.28%)	9 (11.11%)	3 (12.50%)	1 (12.50%)	χ^2^ = 137.3	< 0.0001
MT + IVA	36 (14.63%)	14 (17.28%)	2 (8.33%)	2 (25.00%)		
MT + IVA + PPV	186 (75.61%)	53 (65.43%)	1 (4.17%)	2 (25.00%)		
Enucleation	11 (4.47%)	5 (6.17%)	18 (75.00%)	3 (37.50%)		
COculture(+)*	90 (40.72%)	40 (54.05%)	7 (58.33)	4 (50.00%)		
G+ bacteria	74 (82.22%)	33 (82.5%)	4 (57.14%)	4 (100.00%)	χ^2^ = 40.42	0.0004
G- bacteria	9 (10.00%)	7 (17.5%)	0 (0.00%)	0 (0.00%)		
Mixed bacteria	4 (4.44%)	0 (0.00%)	0 (0.00%)	0 (0.00%)		
Fungus	3 (3.33%)	0 (0.00%)	3 (42.86%)	0 (0.00%)		
**VA**						
pre-therapy	2.22 ± 0.63	2.30 ± 0.52	2.80 ± 0.35	2.44 ± 0.70	F = 7.198	< 0.0001
≦2.3	195 (79.27%)	68 (83.95%)	22 (91.67%)	7 (87.50%)	χ^2^ = 3.307	0.3467
> 2.3	51 (20.73%)	12 (14.81%)	2 (8.33%)	1 (12.50%)		
post- therapy	1.59 ± 0.80	1.53 ± 0.76	2.64 ± 0.69	2.34 ± 0.87	F = 15.82	< 0.0001
≦2.3	78 (31.71%)	21 (25.93%)	19 (79.17%)	6 (75.00%)	χ^2^ = 30.2	< 0.0001
> 2.3	168 (68.29%)	59 (72.84%)	5 (20.83%)	2 (25.00%)		
**VA improved**						
Not improved	80 (32.52%)	15 (18.52%)	19 (79.17%)	6 (75.00%)	χ^2^ = 36.96	< 0.0001
Improved	166 (67.48%)	66 (81.48%)	5 (20.83%)	2 (25.00%)		
Wound location**						
Zone I	197 (80.08%)	–	–	–		
Zone II	44 (17.89%)	–	–	–		
Zone III	5 (2.03%)	–	–	–		
IOFB**						
NO	159 (64.63%)	–	–	–		
Metal	69 (28.05%)	–	–	–		
Non-metal	18 (7.32%)	–	–	–		

*141 cases of causative organisms positive cultures were included in statistics analysis

**246 cases of posttraumatic endophthalmitis were included in statistics analysis

*CO* Causative organisms, *IOFB* intraocular foreign body, *PPV* pars plana vitrectomy, *IVI* intravitreal injection, *MT* medical therapy, *IVA* intravitreal antibiotic injections, *VA* visual acuity, *G+* gram-positive, *G-* gram-negative

Statistical analysis of the factors among the four groups is shown in Table 2. There was no significant difference per year and affected eyes among the four groups (χ^2^ test: *P >* 0.05), but patients with endogenous endophthalmitis are more susceptible to the left eye. Age demonstrated a significant difference among the four groups (F = 43.04, *P* < 0.001), while the posttraumatic (41.67 ± 15.51 years) and the endogenous (67.13 ± 16.13 years) groups had the lowest and highest age, respectively. There were significantly more men (89.02%, *P* < 0.05) in the posttraumatic group compared with the other groups, while the corneal ulcer-associated endophthalmitis tended to affect more women (75.0%) (χ2 = 84.69, *P* < 0.001). There was a significant difference in occupation distribution among the four groups, and the posttraumatic group tended to have more workers (43.90%), while the other groups tended to have more farmers (38.27, 66.67, and 50.00%, respectively, *P* < 0.001). The patient visit time significantly differed among the groups (χ^2^ = 47.41, *P* < 0.0001). Regarding therapy modalities, PPV was the most commonly performed in cases of posttraumatic and postsurgical endophthalmitis, while enucleation accounted for the most in corneal ulcer-associated and endogenous endophthalmitis cases (χ^2^ = 137.3, *P* < 0.0001). The causative organisms presented a significant difference among the four groups (χ^2^ = 40.20, *P* = 0.0004). The most cases of endogenous endophthalmitis tended to be caused by Gram-positive organisms (100%), while fungus was the major cause in the corneal ulcer-associated group (42.86%). The pre-therapy and post-therapy visual outcome were significantly different among the four groups (F = 7.198, F = 15.82, respectively, *P* < 0.0001). The VAs improved and not improved in 120 (33.52%) and 238 (66.48%) cases. The improvement rate of VA was the highest in cases of postsurgical endophthalmitis (81.48%), while the VAs were not significantly improved in cases of corneal ulcer-associated (79.17%) and endogenous (75.00%) endophthalmitis.

### Therapy modalities

The therapy modalities are listed in Table 3. MT, defined as appropriate, topical, periocular, and systemic antibiotic administration, was used in 26 (7.24%) cases. Combined vitrectomy and intraocular antibiotics were used in 243 (67.69%) cases, whereas 54 (15.04%) cases were treated with intravitreal antibiotic injections (IVA) alone, including vancomycin and ceftazidime. Enucleation was performed in 36 cases (10.03%), 10 of which had their eyes removed in a second operation. Corneal ulcer-associated endophthalmitis was the most frequent cause in the enucleation group (Table 2).

**Table 3:** Therapy modalities

Therapy modalities	*n*	Rate (%)
1 MT	26	7.24%
2 MT + IVA	54	15.04%
3 MT + IVA + PPV	243	67.69%
4 Enucleation	36	10.03%
Total	359	100.0%

*MT* medical therapy, *IVA* intravitreal antibiotic injections, *PPV* pars plana vitrectomy

### Visual outcome

Visual outcomes were assessed for 358 eyes, excluding the eye of one child who did not cooperate during the vision test. The average logMAR BCVA value pre-therapy was 2.28 ± 0.60 and significantly improved to 1.67 ± 0.83 at post-therapy (t = 7.161, *P* < 0.0001).

**Fig. 2:**
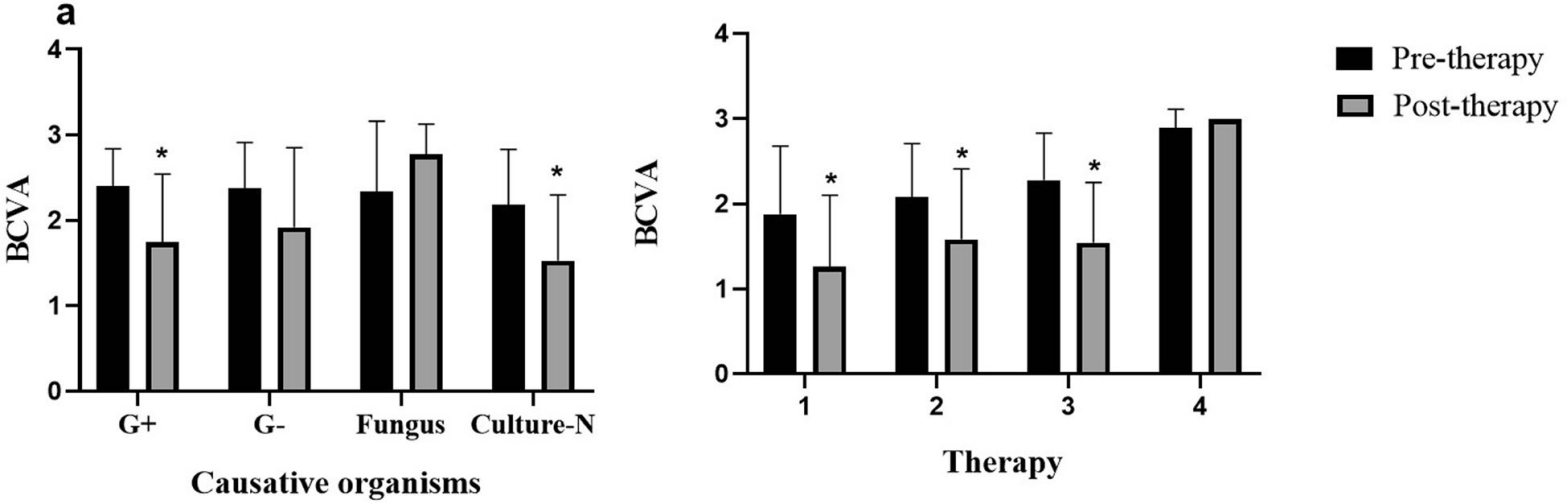
Comparison of pre- and post-therapy BCVA between difference causative bacteria **a** and therapy modalities **b** *Using analysis of variance (ANOVA), *P*-value between pre-therapy BCVA and post-therapy BCVA *P* < 0.05. (1:MT, 2. MT + IVA. 3:MT + IVA + PPV. 4: Enucleation)

Pre-therapy and post-therapy VAs were compared among the four groups (Gram-positive, Gram-negative, fungi, and culture-negative) are shown in Fig. 2a. We observed a significant difference in VA between Gram-positive (G+) and culture-negative patients at pre-therapy and post-therapy (*P* < 0.0001), while there was no significant difference in patients with Gram-negative (G-) bacterial and fungal infection. Figure 2b showed the statistical difference between pre-therapy and post-therapy in MT, MT + IVA, and MT + IVA + PPV (*P* < 0.0001), while the post-therapy VA values were not significantly different in these therapy modalities.

Demographic and clinical features associated with improved VA are demonstrated in Table 4. There were no significant differences in average age, but there were in the segment of age and sex (*P* < 0.05). For eye and occupation, there were no statistically significant differences (*P >* 0.05). The average visit time and time segment were statistically different in the not-improved and improved groups. It was noted that post-traumatic and post-surgical endophthalmitis had obvious improved visual outcomes compared to those having other types of endophthalmitis, and the difference was statistically significant (χ^2^ = 41.98, *P* < 0.0001). For causative organisms, in 141 cases of positive cultures, G+ coccus showed the most favorable visual outcomes when compared to others, presenting statistically significant differences (χ^2^ = 12.93, *P* = 0.0047). Regarding therapy modalities, PPV led to significantly improved visual outcomes (χ^2^ = 87.15, *P* < 0.0001), and the pre-therapy VA displayed an important role in improving vision, especially the patient’s VA ≦logMAR 2.3 showed a trend of statistically significant improvement (χ^2^ = 9.00, *P* = 0.003). Among 246 patients with post-traumatic endophthalmitis, the presence of IOFB (χ^2^ = 4.841, *P* < 0.0001) and the wound location (χ^2^ = 7.398, *P* = 0.0247) were significantly correlated with improved VA. However, there was no significant difference between metal and non-metal IOFB. The visual improvement in zone I was significantly better than that in zone II and III.

**Table 4:** Patient demographic characteristics of multivariable logistic regression analysis of the factors associated with improved visual acuity in 358 cases

Factors	Not improved	Improved	t/χ^**2**^	***P***	Multivariable analysis	
	(120)	(238)			***OR(95%CI)***	***P***
**Age**	47.33 ± 17.65	48.54 ± 18.41	t = 0.595	0.552		
0–15	1 (0.83%)	15 (6.30%)	χ^2^ = 14.30	0.014	0.00 (Reference)	
16–30	24 (20.00%)	23 (9.66%)			0.462 (0.110–1.950)	0.293
31–45	31 (25.83%)	61 (25.63%)			0.434 (0.110–1.726)	0.234
46–60	31 (25.83%)	77 (32.35%)			0.557 (0.143–2.169)	0.399
61–75	25 (20.83%)	40 (16.81%)			0.742 (0.181–3.034)	0.678
> 75	8 (6.67%)	22 (9.24%)			0.400 (0.088–1.813)	0.235
**Sex**						
Male	102 (85.00%)	180 (75.63%)	χ^2^ = 4.188	0.041	0.00 (Reference)	
Female	18 (15.00%)	58 (24.37%)			1.387 (0.719–2.675)	0.329
**Occupation**						
Farmer	50 (41.67%)	91 (38.24%)	χ^2^ = 3.825	0.575	–	
Worker	40 (33.33%)	78 (32.77%)				
Student	4 (3.33%)	17 (7.14%)				
The retired	5 (4.17%)	17 (7.14%)				
Office clerks	6 (5.00%)	9 (3.78%)				
Others	15 (12.50%)	26 (10.92%)				
**Eye**						
Right	58 (48.33%)	118 (49.58%)	χ^2^ = 0.0496	0.824	–	
Left	62 (51.67%)	120 (50.42%)				
**Visit time**	8.86 ± 13.33	5.68 ± 9.10	t = 2.654	0.0083		
≦7D	85 (70.83%)	194 (81.51%)	χ^2^ = 5.290	0.0214	0.00 (Reference)	
>7D	35 (29.17%)	44 (18.49%)			0.522 (0.286–0.953)	0.034
**EC**						
PT	80 (66.67%)	166 (69.75%)	χ2 = 41.98	< 0.0001	0.00 (Reference)	
PS	15 (12.50%)	65 (27.31%)			2.100 (1.109–3.974)	0.023
CA	19 (15.83%)	5 (2.10%)			0.202 (0.066–0.621)	0.005
Ed	6 (5.00%)	2 (0.84%)			0.194 (0.286–0.953)	0.053
**Pre-therapy VA**	2.28 ± 0.60	1.67 ± 0.83	t = 7.161	< 0.0001		
>logMAR2.3	60 (50.00%)	79 (33.19%)	χ^2^ = 9.00	0.003	0.00 (Reference)	
≦logMAR2.3	60 (50.00%)	159 (66.81%)			1.809 (1.052–3.100)	0.032
**TM**						
MD	4 (3.33%)	22 (9.24%)	χ^2^ = 87.15	< 0.0001		
MD + IVI	23 (19.17%)	31 (13.03%)				
MD + IVI+PPV	57 (47.50%)	185 (77.73%)				
Enucleation	36 (30.00%)	0 (0.00%)				
CO Culture(+)*	49	92			–	
G+ bacteria	35 (71.43%)	80 (88.24%)	χ^2^ = 12.93	0.0047		
G- bacteria	7 (14.29%)	9 (8.24%)				
Mixed bacteria	1 (2.04%)	3 (3.53%)				
fungus	6 (12.24%)	0 (0.00%)				
IOFB*						
No	44 (55.00%)	115 (69.28%)	χ^2^ = 4.814	0.0282		
Yes	36 (45.00%)	51 (30.72%)				
Metal	27 (75.00%)	44 (86.27%)	χ^2^ = 1.787	0.1812		
Non-metal	9 (25.00%)	7 (13.73%)				
Wound location*	80	166	χ^2^ = 7.398	0.0247	–	
Zone I	58 (72.50%)	139 (83.73%)				
Zone II	18 (22.50%)	26 (15.66%)				
Zone III	4 (5.00%)	1 (0.60%)				

*246 cases of posttraumatic endophthalmitis were included in the χ^2^ test, but not in the Logistic regression analysis

** 141 cases of positive causative organism’s cultures were included in the in the χ^2^ test, but not the Logistic regression analysis

*TM* Therapy modalities, *EC* Etiological classification, *CO* Causative organisms, *OR* Odds ratio, *PT* Post-traumatic, *PS* Post-surgical, *CA* Corneal ulcer-associated, *Ed* Endogenous

### Binomial logistic regression analysis of predictive factors of improved VA

Multivariate analysis using a binomial logistic regression model was conducted to examine the predictive factors of improved VA. After adjusting for possible confounding factors, visit time > 7 day (*P =* 0.034, OR = 0.522, 95%CI: 0.286–0.953), pre-therapy VA ≦logMAR 2.3 (*P* = 0.032, OR = 1.809, 95%CI: 1.052–3.110), etiology of PS (vs. PT; *P* = 0.023, OR = 2.100, 95% CI: 1.109–3.974) and etiology of CA (vs. PT; *P* = 0.005, OR = 0.202, 95% CI: 0.066–0.621) were significantly associated with improved VA (Table 4).

## Discussion

In our retrospective study, the average age of patients was 48.01 ± 18.27 years, and the patients were mostly young and middle-aged, resulting in a significant impact on their families and our society. Interestingly, the average age of patients with endophthalmitis in Germany was 69.3 ± 1.7 years [14], while the corresponding in western China was 35.1 ± 20.3 years [2]. There were significant differences in sex distribution among the patients with endophthalmitis between the developed and developing countries. In developed countries, the distribution in men and women was almost the balance [3], but in India [16] and China (the western region) [2], the proportion in men was significantly higher than in women, which is consistent with our result. The latter could be explained as women are rarely engaged in physical activity, which results in less ocular trauma. In our study, the patients were farmers (39.3%), workers (32.9%), office clerks (4.2%), retirees (6.1%), students (16.1%), and others (11.4%). The highest proportion of patients consisted of farmers and workers, which was similar to the epidemiological characteristics in other countries [2, 14, 17]. This may be related to the fact that workers and farmers are more prone to trauma and the high-risk nature of their occupations. For injured eyes, there is no statistical difference between the left and right eyes in general.

The results obtained for pathogenic bacteria were consistent with the findings of most reports [2, 14, 18, 19]. We obtained anterior aqueous humor and or vitreous fluid for bacterial culture from 316 patients, and the culture-positive rate was 44.62%. Among the culture-positive cases, Gram-positive bacteria, Gram-negative bacteria, fungi, and mixed bacterial infections accounted for 81.6, 11.3, 4.3, and 2.8% of the cases. *S.epidermidis* was the highest rate in the common pathogens, which was similar with that in Thailand [18]. In contrast, *S. aureus* is more common in other countries [14, 20]. The causative organisms are highly correlated with VA [21]. In terms of the relationship between bacteria and VA, there was a significant difference in the visual acuity between pre-therapy and post-therapy in the Gram-positive and culture-negative groups, but there was no significant difference in the Gram-negative and fungal infection groups, which may be related to the highly virulent and the rapid infection progress of Gram-negative bacteria and the poor therapeutic effect in these cases [12]. Thus, the pathogenicity of the microorganisms significantly influenced the prognosis.

The etiology of the disease was categorized into four groups: posttraumatic, postoperative, corneal ulcer-related, and endogenous [22]. We analyzed the various factors’ differences among the four groups. In our study, posttraumatic endophthalmitis was the main cause, accounting for 68.52%, followed by postoperative complications (22.56%). Similarly, a German report showed that endogenous endophthalmitis accounted for 41% of the cases [9]. In contrast, a study conducted in Odisha, India, reported that 43.0% of the cases had a postoperative etiology while 40.2% were post-traumatic [16], while one study performed in the western region of China reported that 82.6% of 1593 endophthalmitis cases were posttraumatic. In the posttraumatic group, the patients were significantly younger with an average age of 41.67 ± 15.51 years. The average logMAR BCVA significantly improved from 2.22 ± 0.63 on pre-therapy to 1.59 ± 0.80 on post-therapy (*P* < 0.0001), the VA improvement rate reached 67.48%, which might be related to PPV, the main therapy modality. Regarding the pathogenic bacteria, the Gram-positive bacteria were the most common and even multiple mixed infections existed [23]. Wound location was dominated by zone I, and 87 cases (35.4%) were complicated with intraocular foreign bodies, including 71 (28.5%) and 16 (6.5%) cases of metallic and non-metallic foreign bodies, respectively, in line with findings from other regions [23, 24]. Regarding the postoperative group, the proportion of retirees and the age increased significantly, with an average age of 62.21 ± 17.17 years, and there was no significant difference in the male to female ratio, and the VA improvement rate (81.48%) was the most significant among the four groups, while the treatment was also dominated by PPV. However, in the corneal ulcer-related endophthalmitis group, the proportion of women was the highest among the groups, and the pathogenic bacteria were G+ bacteria and fungi; because of the delayed visit time and the poor pre-therapy VA, the proportion of enucleation was the highest in the four types. In endogenous endophthalmitis, which was caused by hematogenous spread, we did not only find that the average age was the oldest and the pathogenic bacteria were all G+ bacteria, which was consistent with previous reports [7, 21]; interestingly, the left eye was most involved, and there was the statistical difference compared with the posttraumatic and postoperative groups. This finding was inconsistent with the previous findings that reported greater involvement of the right eye than of the left eye [3, 25]. We hypothesized that a bacterial embolus is more likely to enter the left carotid artery and flow into the terminal artery, the central retinal artery of the left eye, considering the anatomy character, the right common carotid originates in the neck from the brachiocephalic trunk, while the left directly arises from the aortic arch. Nevertheless, this result may not be convincing, due to the small sample sizes and the limitations of ophthalmology hospitals. Different etiology, different VA prognosis results, and corneal ulcer-related endophthalmitis had the worst postoperative VA (2.64 ± 0.69). The posttraumatic and postoperative groups presented significantly improved VAs, while corneal ulcer-related and endogenous endophthalmitis had a higher rate of unimproved VA, with statistically significant differences.

All 359 patients received topical and intravenous antibiotics, including vancomycin alone or ceftazidime in combination. All patients did not receive intraocular antibiotics before onset; however, some patients underwent systemic antibiotic administration [23]. There are different therapeutic modalities for infectious endophthalmitis according to the severity. According to our data analysis, there was a relationship between different therapy and vision improvement. According to Fig. 2 and Table 4, in addition to enucleation, the improvement of VA was statistically significant in various treatment methods, while in the PPV group this improvement was significantly higher than in the other groups, which was similar to the results of other researches [4, 6]. Therefore, PPV is considered the main therapy strategy in cases of infectious endophthalmitis [4, 5, 22].

The improved VA after treatment was considered the primary outcome, therefore we investigated various factors that may affect the improvement of VA, including age, sex, etiology, pre-therapy VA, patient visit time, pathogenic microorganisms, therapy modalities etc. In the posttraumatic endophthalmitis, the presence of IOFB and wound location were associated with improved vision, but the metal or non-metal relationship was not significant. The improvement in vision in zone III was significantly worse than in zone I and II. Finally, multivariate analysis using binomial logistic regression was performed on these factors with *P >* 0.05, while the therapy modalities were an intermediate variable, affected by many factors, so they were not included in the regression study. We found that the patient visit time, pre-therapy VA, and the etiology were important factors for visual improvement, and no correlation was found with age and pathogenic bacteria. The visual outcomes caused by different pathogenic bacteria were inconsistent, but no statistical difference was found in logistic regression analysis, which may be caused by a relatively lower bacterial positive culture rate, correlations with etiology, patient visit time, and visual outcome.

Nevertheless, this retrospective analysis had some limitations: (1) It had a single-center and retrospective design. A prospective, randomized study design would have been more desirable. (2) The Shanxi Eye Hospital is the largest ophthalmology hospital in Shanxi Province. However, patients with a severe systemic disease are referred to a general hospital; thus, we may not have obtained the data for a large number of patients with endogenous endophthalmitis, leading to sample bias. (3) In terms of VA, we did not perform final follow-up assessments, and we only assessed the vision at a single hospitalization and discharge, because traumatic cataract and postoperative inflammatory reaction would affect vision. In future studies, we intend to collect these data. Therefore, we will perform longer follow-up time studies in the future to observe more potential complications and final recover sight. Moreover, multiple imaging modalities (e.g., structural and functional) optical coherence tomography, fluorescein angiography, indocyanine green angiography, and autofluorescence can be included to find more quantitative parameters in patients diagnosed with infectious endophthalmitis.

## Conclusions

In summary, our study revealed that the incidence of infectious endophthalmitis was much higher in farmers and workers of middle age population in Shanxi Province. Moreover, the final visual outcomes were related with types of endophthalmitis, and two additional factors, such as early treatment and good initial visual acuity, played an important role in the final visual recovery.

## Data Availability

The analytical data in this study could be obtained from the corresponding author upon reasonable request.

## Abbreviations

BCVA: Best-corrected visual acuity
CA: Corneal ulcer-associated
CFs: Counting fingers
CO: Causative organisms
EC: Etiological classification
Ed: Endogenous
G-: Gram-negative
G +: Gram-positive
HM: Hand movement
IOFB: Intraocular foreign body
IVA: Intravitreal antibiotic injections
IVI: Intraocular injection
LP: Light perception
MT: Medical therapy
NLP: No LP
OR: Odds ratio
PPV: Pars plana vitrectomy
PS: Post-surgical
PT: Post-traumatic
TM: Therapy modalities
VA: Visual acuity
